# Multi-center evaluation of radiomics and deep learning to stratify malignancy risk of IPMNs

**DOI:** 10.21203/rs.3.rs-6622868/v1

**Published:** 2025-05-30

**Authors:** Andrea M. Bejar, María Jaramillo Gonzalez, Ziliang Hong, Gorkem Durak, Elif Keles, Halil Ertugrul Aktas, Zheyuan Zhang, Hongyi Pan, Zeynep Sue Jozwiak, Fergan Bol, Lili Zhao, Chao Chen, Concetto Spampinato, Alpay Medetalibeyoglu, Sukru Mehmet Erturk, Gulbiz Dagoglu Kartal, Yury Velichko, Emil Agarunov, Ziyue Xu, Sachin Jambawalikar, Ivo G. Schoots, Marco J. Bruno, Chenchang Huang, Tamas Gonda, Candice Bolan, Frank H. Miller, Michael B. Wallace, Rajesh N. Keswani, Pallavi Tiwari, Ulas Bagci

**Affiliations:** Northwestern University; University of Wisconsin-Madison; Northwestern University; Northwestern University; Northwestern University; Northwestern University; Northwestern University; Northwestern University; Northwestern University; University of Health Sciences Istanbul Bakirkoy Dr. Sadi Konuk Training and Research Hospital; Northwestern University; Stony Brook University; University of Catania; Istanbul University Faculty of Medicine; Istanbul University Faculty of Medicine; Istanbul University Faculty of Medicine; Northwestern University; New York University; NVIDIA; Columbia University in the City of New York; Erasmus University Medical Center; Erasmus Medical Center; New York University; New York University; Mayo Clinic; Northwestern University; Mayo Clinic; Northwestern University; University of Wisconsin-Madison; Northwestern University

## Abstract

Distinguishing high-risk intraductal papillary mucinous neoplasms (IPMNs), pancreatic cysts requiring surgery, from low-risk lesions remains a clinical challenge, often resulting in unnecessary procedures due to limited specificity of current methods. While radiomics and deep learning (DL) have been explored for pancreatic cancer, cyst-level malignancy risk stratification of IPMNs remains untapped. We conducted a multi-institutional study (seven centers, 359 T2W MRI images) to assess the feasibility of AI for predicting IPMN dysplasia grade using cyst-level image features. We developed and compared 2D and 3D radiomics-only, deep learning (DL)-only, and radiomics-DL fusion models, using expert radiologist scoring as a baseline reference. Model performance was evaluated using held-out test data. The radiomics-DL fusion model showed the highest discriminatory ability on the test set (AUC 0.692), outperforming the radiomics-only model (AUC 0.665). Expert accuracy varied widely (37.4%-66.7%). The fusion model integrating deep learning and radiomics features from routine T2W MRI (AUC: 0.692) demonstrates potential for objective, cyst-level risk stratification of IPMNs in a multi-center cohort, outperforming both radiomics-only models and expert radiologists. While performance requires improvement for standalone clinical use, this approach offers a scalable, non-invasive method to potentially improve diagnostic accuracy and reduce unnecessary surgical interventions.

## INTRODUCTION

The increasing detection of *pancreatic cysts* has become a significant clinical challenge^1-3^, imposing a substantial burden on patients (due to invasive procedures and surgical risks) and healthcare systems (due to cost of surveillance and interventions). Intraductal papillary mucinous neoplasms (IPMNs), a potentially premalignant cyst subtype, constitute a substantial proportion (estimated 50–80%) of these incidental lesions^1-3^. Despite their premalignant potential, the risk of malignant transformation in IPMNs remains poorly defined. Resection studies report a wide range of malignancy rates of 1–38% for branch duct (BD) IPMN and 33–85% for main duct (MD) IPMN; figures that likely overestimate the true rate of progression^2,3^. The most recent international consensus guidelines, the *2023 Kyoto criteria*, represent the current standard for IPMN management^2^. However, the limitations in accurately diagnosing pancreatic cystic disease and assessing the risk of malignancy in pancreatic cysts according to existing guidelines continue to impose a substantial burden on patients and healthcare systems. These shortcomings frequently lead to invasive diagnostic procedures and high-risk surgical resections, especially for lesions ultimately deemed low-grade^4-9^.

Magnetic resonance imaging (MRI) and endoscopic ultrasound (EUS) with fine needle aspiration (FNA) are primary modalities for IPMN diagnosis and characterization^2^. EUS-FNA is particularly valuable for evaluating pancreatic cysts and assessing IPMN dysplasia grade. Accurate IPMN characterization prior to invasive procedures is necessary to lower patient burden and cost. While EUS-FNA procedures carry a low complication rate (up to 3%) and rare mortality, the diagnostic sensitivity of EUS-FNA histopathology remains limited, ranging from 4.8–61.6%^10-12^. Thus, even after this invasive, operator-dependent, and costly procedure, malignancy cannot be reliably excluded, leaving patients at risk of bleeding, pancreatitis, and infection^2,6,10,11^. A leading indication for the surgical resection of cystic lesions is a concern for malignancy^13^. However, pancreatic resections are major surgeries with significant morbidity and mortality rates and as a result it is critical to diagnose suspicious lesions prior to surgery^14,15^.

Radiomics, leveraging high-throughput quantitative image analysis, enables the extraction and analysis of quantitative features imperceptible to the human eye^16^. Deep learning (DL), a neural network-based advanced artificial intelligence (AI) technique, utilizes convolution to effectively extract and discern complex imaging patterns^17^. While radiomics and DL have advanced pancreatic tumor detection and segmentation in computed tomography (CT) and MRI, their application to characterization of premalignant lesions including IPMNs remains nascent^18^. Recent studies propose radiomics, DL, or fused models for IPMN diagnosis and classification^19-23^; however, critical barriers persist. First, the pancreas’ retroperitoneal anatomy and heterogeneous parenchyma complicate image analysis^18^. Second, IPMNs exhibit marked variability in morphology and texture, even within individual cysts^18^. Third, DL demands large, diverse datasets, yet pancreatic MRI—the optimal and preferred modality for cyst characterization—remains scarce and protocol-dependent^3,18,24-26^. Prior studies using radiomics/DL have focused predominantly on tumor detection or whole-pancreas analysis, potentially overlooking crucial information within the heterogeneous cyst itself ^18^. Many analyses exclude higher-risk MD/mixed-IPMNs, limiting applicability to the full spectrum of disease. Furthermore, smaller single-center cohorts (< 150 patients) limit generalizability^19-22^. Our work addresses these gaps by performing a large, multicenter evaluation focused specifically on cyst regional level features from T2W MRI, including MD- and mixed-type IPMNs, to predict dysplasia grade using 2D and 3D radiomics, DL, and fusion approaches.

## RESULTS

In this section, we evaluate the development and performance of three advanced machine learning algorithms for the stratification of IPMN dysplasia grade in MRI: 1) radiomics-only; 2) DL-only; 3) radiomics-DL fusion. Each approach was assessed using rigorous validation protocols across our multicenter dataset.

### Evaluation of dataset heterogeneity using UMAP

**Figure 1:**
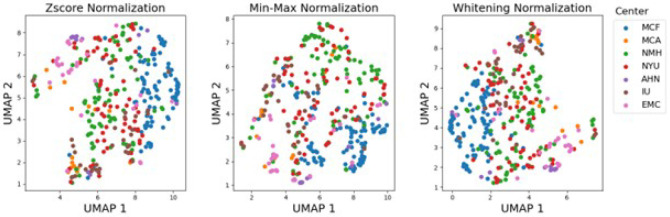
UMAP of quality indicators (projected into x and y axes from 21 quality indicators) per center using different normalization methods. Centers: Mayo Clinic Florida (MCF), Mayo Clinic Arizona (MCA), Northwestern Memorial Hospitals (NMH), New York University (NYU), Allegheny Health Network (AHN), Istanbul University (IU) Hospital, and Erasmus Medical Center (EMC).

A UMAP (uniform manifold approximation and projection) was used to explore high-dimensional representation of the multi-institutional MRI data using the normalized image quality indicators is shown in Fig. 1. UMAP revealed distinct clusters of scans associated with different centers (a total of seven) and directly related to voxel height of MRI images. One cluster comprised scans primarily from the Mayo Clinic Florida (MCF) center (blue), characterized by a mean voxel height of 4 mm. Another cluster included scans from the Northwestern Memorial Hospitals (NMH) and New York University (NYU) clinical centers (green and red), with respective mean voxel heights of 5.5 mm and 5 mm. A separate cluster consisted of Erasmus Medical Center (EMC) scans (pink), notable for a 7.3 mm mean voxel height and acquisition exclusively on a 1.5T MRI magnet. Scans from the MCA, Allegheny Health Network (AHN), and Istanbul University (IU) Hospital centers (orange, purple, and brown), exhibiting a 7 mm mean voxel height, were distributed outside these primary clusters. These findings underscore significant variations in image quality across participating centers.

### Manual segmentation of index cystic lesions

**Figure 2:**
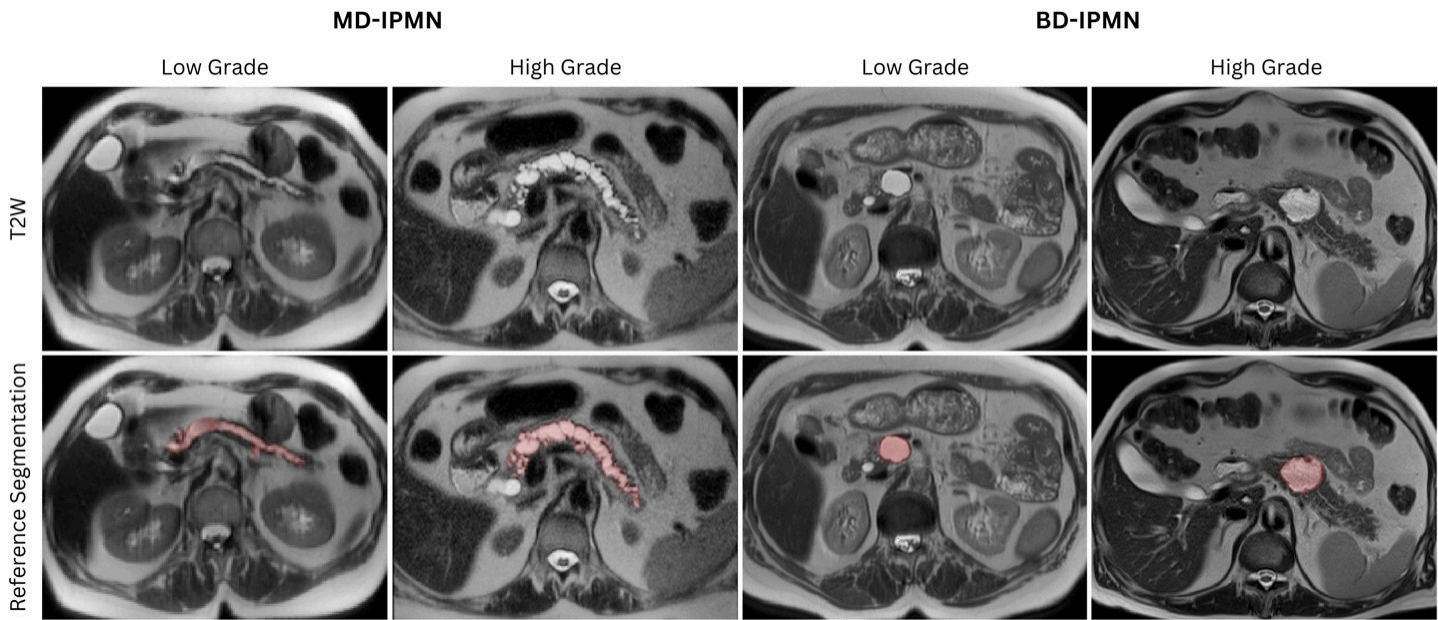
Representative T2-weighted (T2W) images and reference segmentations of high-grade and low-grade IPMNs. The first row shows T2W MRI images, and the second row shows reference segmentations of high-grade and low-grade, main-duct (MD)-IPMN and branch-duct (BD)-IPMN cases.

Intraobserver mean dice similarity coefficient (DSC) across three readers (abbreviated as GK, AMB, and HEA) was 80% and a Hausdorff Distance at 95 percentile (HD95) of 6.63 mm. Interobserver mean DSC was 75% with a HD95 of 7.2 mm. These DSC and HD95 values are indicative of high segmentation consistency and support the reliability of our reference standard segmentations. Representative T2-weighted (T2W) MRI images and corresponding segmentations of main-duct (MD) and branch-duct (BD)-IPMN across varying dysplasia grades are shown in Fig. 2.

### Visual scoring and risk prediction

Diagnostic performance metrics are summarized in Table 1. Sensitivity analysis revealed that Rater 3 achieved the highest detection rate (72.3%, 95% CI: 64.1–79.5%), while Rater 1 demonstrated superior specificity (64.5%, 95% CI: 57.8–70.9%). In cases with concordant majority readings (n = 337), positive percent agreement was calculated. Pairwise comparisons of overall accuracy demonstrated significant heterogeneity, with the highest concordance observed between Raters 1 and 3 (80.1%, p < 0.001), and the lowest between Raters 1 and 2 (48.1%, p = 0.042). Cohen's kappa coefficient analysis indicated fair to moderate agreement between raters with κ of 0.33 to 0.67, as detailed in Table 2. The inter-observer variability demonstrated statistically significant heterogeneity (Cochran's Q test, p < 0.001), underscoring the subjective nature of visual assessment in this context.

### Prediction using 2D and 3D radiomic features

**Figure 3:**
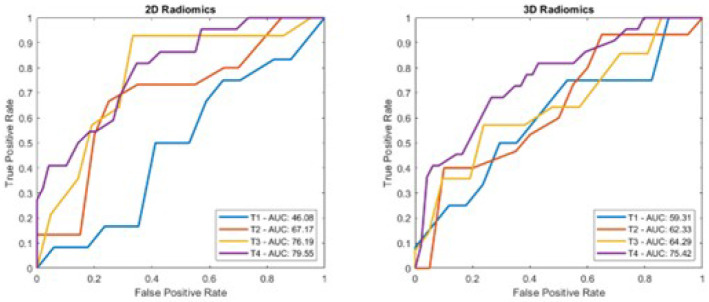
Receiver operating characteristic curve of 2D and 3D radiomics predictions distinguishing between Low and High-Risk groups in the testing set.

**Figure 4:**
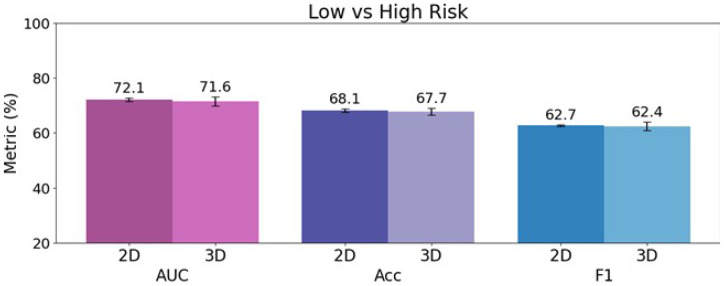
Low vs High Risk classification comparison in cross-validation set’s mean (%) AUC, accuracy, and F1 for 2D and 3D radiomic analyses. Means are written above the error bars, and error bars show standard deviation.

**Figure 5:**
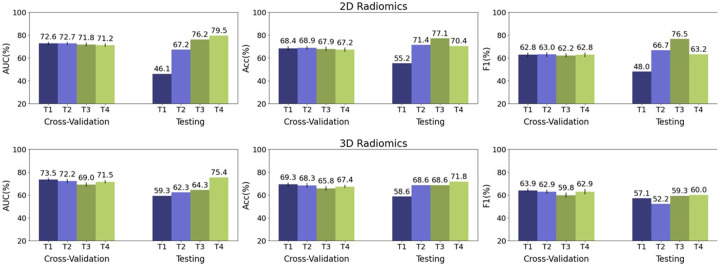
Low vs High Risk classification comparison of mean (%) AUC, accuracy, and F1 between cross-validation and testing trials for 2D and 3D radiomic analyses. Values above each bar represent the mean value. Error bars show standard deviation.

The 2D radiomic analysis yielded a mean AUC of 66.4% with mean accuracy of 65.9%. The 3D analysis yielded a mean AUC of 66.5% and a mean accuracy of 66.1% (Table 3). The corresponding ROC curves are shown in Fig. 3. Bar plots displaying the individual and mean testing set AUC, acc, and F1 are shown in Fig. 4 and Fig. 5, respectively.

Considering expert-judgment results (radiologists’ scoring based on imaging features of the Kyoto guidelines) in previous section, radiomics results are shown to be superior to two of three radiologists and performing on par with the majority-voting based results. It should also be noted that radiologists used both T1W and T2W scans and Kyoto guidelines for determining the cysts stratification while our DL and radiomics analysis used only T2W, indicating the promising and superiority nature of machine generated results.

### Comparing the performance of six DL architectures in predicting IPMN dysplasia grade

Among the tested various CNNs, DenseNet121^27^ demonstrated the highest AUC at 73.3% (Table 4). In comparison, ResNet-34^28^ achieved a slightly lower AUC of 73.1%. Lightweight models, such as EfficientNet-B0^29^ and ShuffleNet-V2^30^, exhibited demonstrably lower AUC values of 68.1% and 66.1%, respectively.

### Evaluation of 2D and 3D radiomics-DL fusion algorithms

Using 2D radiomic features, the fusion model achieved a weighted average AUC of 74.3% and an accuracy of 71.0% in cross-validation. In independent testing, this 2D feature fusion model yielded an AUC of 69.2% and an accuracy of 61.6% (Table 5). When trained with 3D radiomic features, the fusion model demonstrated a weighted average AUC of 73.4% and an accuracy of 98.4% in cross-validation, and an AUC of 68.3% and accuracy of 62.7% in independent testing.

## DISCUSSIONS

In this large, multicenter study focused on cyst regional-level IPMN analysis from T2W MRI scans of 359 subjects, we demonstrated the feasibility of using radiomics and DL approaches for malignancy risk stratification of IPMN lesions. Visual scoring, the current standard in clinics, raters had minimal to moderate agreement with weighted Kappa scores of 0.33–0.67^32^. The visual scoring accuracy for the majority cases and Rater 1 were similar to the accuracies of the radiomics-only algorithms on testing; and higher than the accuracies of the fusion algorithms on testing, Rater 2, and Rater 3. Our DenseNet121 deep learning model achieved the highest performance (AUC 73.3%, accuracy 68.0%), followed closely by our radiomics-deep learning fusion algorithm using 2D radiomic features (AUC 69.2%, accuracy 61.6% in testing; AUC 74.3%, accuracy 71.0% in cross-validation). This performance effectively balanced parameter efficiency and predictive power. Lightweight models, EfficientNet-B0 and ShuffleNet-V2, exhibited lower AUC values, underscoring the trade-off between model complexity and predictive accuracy across diverse architectures. The fusion of DL and radiomics algorithm, utilizing 2D radiomic features, attained a weighted average AUC of 69.2% and accuracy of 61.6% in testing, and a weighted average AUC of 74.3% and Acc of 71.0% on cross validation. Radiomics-only analyses, employing 3D features, followed with respective AUC and accuracy of 66.5% and 66.1% on testing. Comparable performance was observed between algorithms utilizing 2D versus 3D radiomic features, indicating the potential utility of computationally efficient 2D methods. Importantly, these advanced methods demonstrated performance that matched or exceeded expert radiologist assessment, highlighting their potential to augment clinical decision-making in IPMN management.

In our earlier work (Yao et.al. 2023), we classified IPMN malignancy risk using advanced analysis techniques coupled with an automatic whole pancreas segmentation algorithm in 246 T1W and T2W MRI scans from five centers^23^. In that work, we developed three algorithms with incorporated clinical features (age, gender, BMI, diabetes mellitus, and chronic pancreatitis) to accomplish this task: a radiomics-only, DL-only, and DL-radiomics fusion using four CNNs and Vision Transformer (ViT). Our algorithms stratified cases as healthy (n = 70), low-grade risk (n = 85), and high-grade risk (n = 91). In our current study; hence, our results are not entirely comparable with Yao et al. 2023^23^ because we switched into two class-classification from three-class classification by focusing only in cystic cases. In our earlier results, we found a mean HD95 of 26.08 mm and DSC of 70.11 for our automatic segmentations that might have introduced additional errors in radiomics and DL analysis. On the other hand, herein, we used manual segmentation (i.e., ground truths) by interdisciplinary experts that were then reviewed by expert radiologists to ensure accurate segmentation; hence, we minimized segmentation induced errors in radiomics and DL analysis. Another key difference compared to our earlier study is our earlier study did not include cyst-type, and all the experiments conducted on a much smaller cohort.

Cui et.al. 2021 conducted a study to develop a nomogram to predict the pathological grade of BD-IPMN^33^. The nomogram incorporated clinical features (sex, symptoms, age, CA19-9, and CEA) and radiomic features derived from manually segmented cysts. Their dataset included T2W, T1W, and contrast enhanced T1W scans pertaining to 202 patients collected from three centers. Their data was classified by dysplasia grades as low or high. In their results, it was found that 24.8% of their BD-IPMN cases had high grade dysplasia. On testing using radiomic-only features, they had specificity, sensitivity, and AUC of 81.6%, 70.0%, and 81.1% respectively on validation. Once radiomic and clinical features were incorporated, their nomogram achieved specificity, sensitivity, and AUC of 79.0%, 90.0%, and 88.4% in validation. To compare our studies, the main difference is the type of IPMN cysts that they have included: BD-IPMN while we utilized MD-IPMN, BD-IPMN and mixed-types. While promising, nomograms may perform poorly when applied to populations different from their development cohort, limiting their generalizability across diverse clinical settings^34^. Additionally, the focus on only BD-IPMN could lead to selection bias in their study because BD-IPMN has a lower risk of malignancy. Their ratio of high-grade dysplasia cases is lower than ours and may not be representative of a real-world cohort of IPMN which we tried to approximate. Furthermore, authors included an additional scan of contrast enhanced T1W sequences in their analysis while we confined ourselves into conventional T1W and T2W. In comparing our results, their radiomics-only analysis outperformed ours in AUC and specificity. This could be highly likely because their analysis included several clinical features like patient symptoms and tumor markers which are known to be predictive of higher risk IPMN^2^. We are aware of the significance of clinical features in predicting IPMN malignancy risk and plan to incorporate them into our future analyses. Despite this, our radiomics-only analysis had similar sensitivity to theirs. This is in spite of our use of multiple centers but could have been due to our larger data set. Overall, our study was done in more of a medical image analysis environment than theirs and could provide a more robust malignancy risk prediction method, and having a promise of even better predictions once other clinical markers are combined with imaging.

To our knowledge, majority of studies that have used radiomics to classify IPMN are largely CT-based^35-38^. MRI is the preferred imaging method for IPMN classification and monitoring compared to CT because it has no radiation exposure, has higher contrast resolution, and it is better at assessing tissue and cysts^3,39^. Furthermore, ours is the most comprehensive study on IPMN malignancy risk stratification that utilizes cyst masks in MRI^20,33,40^. Cheng et.al. 2022 found superior performance of an MRI radiomics algorithm when compared to CT in predicting IPMN malignant potential^20^. Among studies that have utilized MRI, two analyzed only BD-IPMN and the remainder did not specify IPMN type^20,23,33,40^. We found that 38.5% of our Mixed/MD-IPMN and 78.2% of BD-IPMN lesions were Low-Risk. This suggests that many pancreatic resections are unnecessarily performed because a lesion is a MD-IPMN, without any further analysis to stratify lesions that may actually be at risk of malignancy. MD-IPMN is frequently surgically resected in patients that do not have contraindications to surgery, as it has a higher risk of malignant transformation than BD-IPMN^2,3^. Studies that only include BD-IPMN are excluding an important and under-investigated subtype. We included MD- and mixed-IPMN in our advanced algorithm training to approximate a more real-world cohort.

Our study has several limitations that should be considered. First, its retrospective design inherently limits causal inference and introduces potential biases in the historical data collection. Second, the data collected over two decades contributed to variations, including differences in scan quality and uncertainties regarding the accurate grading of dysplasia. The experience levels of operators and pathologists varied across cases, potentially affecting the reliability of dysplasia assessments. Additionally, there was no standardized protocol for selecting cases for EUS-FNA, which may introduce bias since some patients might have undergone EUS for reasons unrelated to the malignancy risk of cystic lesions. Consequently, cytology might have been obtained from cysts that were not classified as high-risk based on imaging. Moreover, the appearance of cysts may have changed in MRI images taken after the EUS procedure, which could complicate image analysis. Despite the risk of cyst appearance changes following EUS, we have found our results to be reliable using segmentations of visible cystic lesions. Acknowledging these concerns, we thoroughly reviewed the dataset to ensure its suitability for the study.

Third, for the BD-IPMN group, we exclusively analyzed data from the sampled cysts. This intentional selection introduced some selection bias; however, focusing on patients at higher risk for malignancy was crucial. Consequently, our observed rates of malignancy risk for BD-IPMNs are similar to higher than those reported in the broader literature, which frequently includes milder cases^2,3^.

Fourth, our dataset was collected from seven institutions using various brands of MRI scanners and field strengths (1.5T and 3T) with differing image acquisition protocols. This variability poses analytical challenges and ultimately affects the algorithm's performance. Although our multicenter dataset is diverse and heterogeneous, this variety strengthens the algorithm's robustness, ensures its stability across different environments, and enhances its applicability in real-world clinical settings, where imaging protocols frequently vary.

Fifth, our image analysis was limited to T2W MRI sequences due to data availability constraints. We plan to include and analyze additional MRI sequences in our future studies. Lastly, radiologist raters utilized only T1W and T2W sequences for expert risk assessment; however, these sequences alone are insufficient for a thorough visual evaluation and do not represent real-life assessments fully. Moreover, the radiologist raters lacked access to previous scans, clinical information, or other critical MRI sequences—such as diffusion sequences—that are valuable for accurately estimating risk. These factors could affect the accuracy of visual scoring compared to standard comprehensive imaging analyses.

These limitations point to several promising directions for future research. Prospective validation studies with standardized imaging protocols would strengthen evidence for clinical translation. Integration of clinical parameters (age, symptoms, tumor markers) and additional MRI sequences (contrast-enhanced, diffusion-weighted) could further improve model performance. Development of ensemble approaches that combine imaging features with other biomarkers (cyst fluid analysis, circulating markers) might provide more comprehensive risk assessment. Finally, extending these methods to predict long-term outcomes rather than cross-sectional histopathology would better align with the clinical goal of identifying lesions likely to progress to malignancy.

In conclusion, our multicenter, pancreatic cyst-focused study demonstrates the feasibility and potential clinical utility of radiomics and deep learning for IPMN risk stratification using routinely acquired T2W MRI scans. While predictive performance requires further enhancement, potentially through integration of clinical data and additional imaging sequences, our advanced machine learning models achieved performance comparable and even better to expert radiologists in this challenging cohort, offering greater objectivity and reproducibility compared to visual assessment. Given that current international consensus guidelines lack optimal specificity for identifying low-risk IPMNs without invasive procedures, computational tools like ours represent a valuable step toward more precise patient selection for intervention versus surveillance. Hence, our findings have immediate clinical relevance. The fusion model's performance, comparable to expert radiologists, suggests potential for integration into clinical workflows as a decision support tool. By providing objective risk stratification of IPMNs, our approach could reduce the high rates of unnecessary surgical resections of low-risk lesions, particularly for MD-IPMNs which are often resected based solely on morphology. Implementation could take the form of a software plugin for radiology workstations, offering real-time risk assessment during routine reads without disrupting workflow. Cost-effectiveness analyses and prospective validation would be logical next steps toward clinical translation.

## METHODS

### Data collection and subject selection

**Figure 6:**
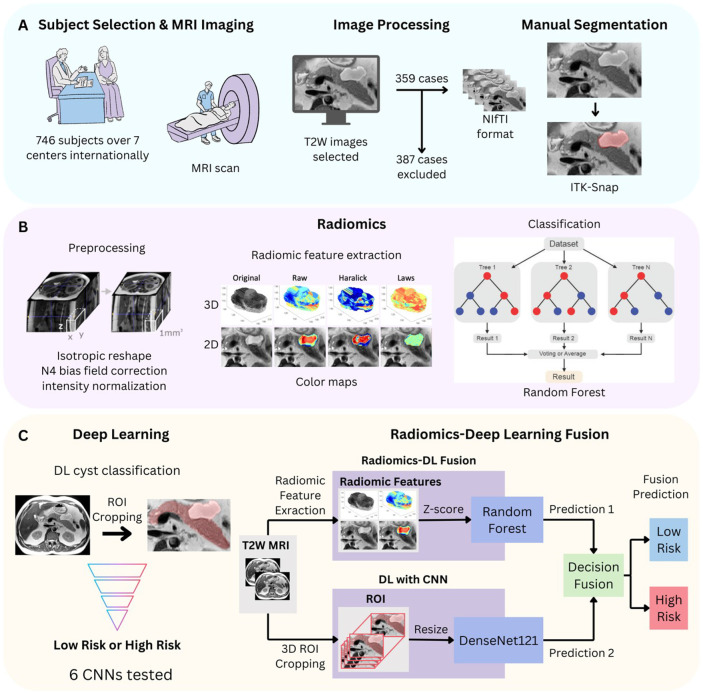
Diagram of patient selection, data set curation, and radiomics and deep learning (DL) pipelines. (A) 746 patients were selected and received MRI imaging from seven centers between three countries; images were then preprocessed and manually segmented. (B) 2D and 3D radiomic features were extracted and classified using a random forest algorithm. (C) A DL-only analysis was conducted, then we developed a radiomics-DL fusion algorithm^27^.

**Figure 7:**
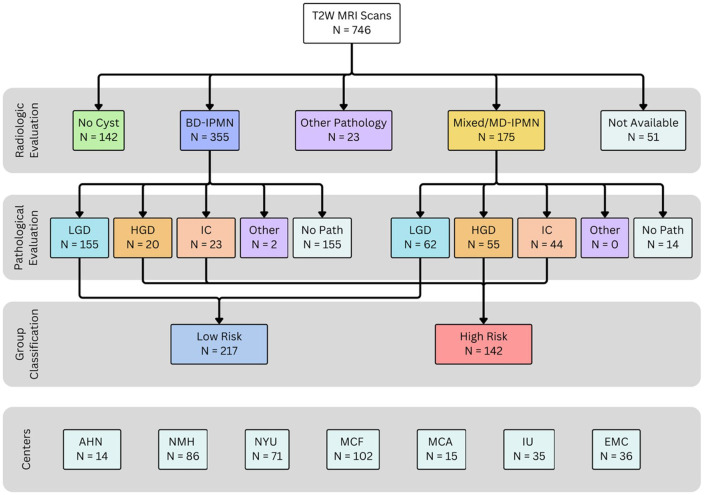
Flowchart of the subject selection and classification.

Our retrospective study (overview in Fig. 6) was approved by an Institutional Review Board (IRB) and all images were de-identified prior to usage in accordance with ethical standards. We collected 746 T2W MRI scans from patients over 18 years of age undergoing assessment for pancreatic cystic lesions between March 2004 and June 2024. Scans were collected from seven centers: Allegheny Health Network (AHN), Erasmus Medical Center (EMC), Istanbul University (IU) Hospital, Mayo Clinic Florida (MCF), Mayo Clinic Arizona (MCA), Northwestern Memorial Hospital (NMH), and New York University Langone Hospital (NYU) (Fig. 6-A). From an initial cohort of 746 subjects, 359 met these inclusion criteria and were selected for analysis (Fig. 7). The selected cohort had a mean age of 67.2 ± 10.8 years and was 53% female. Images were acquired on Siemens, Philips, or GE scanners with either 1.5 T or 3 T field strength. After collection, images were selected converted to Neuroimaging Informatics Technology Initiative (NIfTI) format for analysis. We selected axial, non-fat-suppressed T2W scans; slice thicknesses of original DICOM files were between 3–8 mm and voxel heights of the converted NIFTI files were 3–15.9 mm. This data set includes abdominal MRIs of subjects with pancreatic cysts that were selected from an extended version of the *PanSegNet dataset* by our multicenter group (Zhang et.al., 2025^25^).

We evaluated the radiologic and histopathology results of all subjects prior to inclusion in our study (Fig. 7). On radiologic evaluation, 216 scans were excluded due to the absence of a pancreatic cyst, the presence of a different histopathology, or an unavailable radiologic result. The remaining 530 were further evaluated histopathologically via EUS-FNA or surgical resection. Among this cohort, 171 either did not undergo intervention or had histopathology findings negative for IPMN, leaving 359 patients for our study.

### Subject classification

Dysplasia grades for BD-IPMN were determined via histopathology from EUS-FNA or surgical resection. All MD or Mixed IPMN were surgically resected and histopathologically evaluated. Subjects were then grouped based on dysplasia grade: lesions with low grade dysplasia (LGD) as Low Risk, and lesions with high grade dysplasia (HGD) and/or invasive carcinoma (IC) as High Risk (Table 6). The Low-Risk group was composed of 217 scans, which included 78.2% of the BD-IPMN cases and 38.5% of the MD or mixed-type IPMN cases. The High-Risk group included 142 subjects in total, 75 with HGD and 67 with IC. The High-Risk group included 27.7% of the BD-IPMN cases and 61.5% of the MD or mixed-type IPMN cases. One-third of Low-Risk patients had MD or mixed IPMN and were therefore unnecessarily resected due to current guidelines largely suggesting surgical resection for MD lesions^2^.

### Image quality assessment

To carefully investigate the retrospective data that was accrued across a 20-year time span (2004–2024) from across different institutions, quality indicators were calculated to evaluate center variabilities caused by imaging devices and acquisition protocols. A total of 21 image-quality indicators including statistical values of intensities (e.g. mean, range, variance), and second-order statistics or filter-based measures (e.g. contrast per pixel, entropy focus criterion, and signal-to-noise ratios) were calculated using the open-source MRQy tool^41^. Then, to visualize the quality indicators, the features are projected into a 2D plot using Uniform Manifold Approximation and Projection (UMAP) for Dimension Reduction. Before UMAP projection, each feature was normalized across the dataset using three different methods: z-score, minmax, and data whitening.

### Manual segmentation

The index lesion for each MRI scan was segmented manually and reviewed by an interdisciplinary team of radiologists (GD, an abdominal radiologist with seven years of experience; and FB, a general radiologist with four years of abdominal radiology experience) and students (AMB and HEA, third year medical students; and ZSJ, a fourth year undergraduate student) using ITK-Snap (Version 4.2.0)^42^. All segmentations were reviewed by three expert abdominal radiologists [GD, FB, YBT] to ensure accuracy and consistency. For subjects with BD-IPMN, the index lesion was defined as the cyst that was sampled in EUS-FNA or that was surgically resected. For mixed and MD-IPMN subjects, all regions with cystic involvement were surgically resected. Therefore, approximated cyst boundaries were discussed between abdominal radiologists and decided in consensus prior to segmentation. Concomitant cystic lesions were not included in the analyses.

Interobserver and intraobserver agreements were assessed to evaluate the quality and reproducibility of image segmentations. These are calculated using the Dice Similarity Coefficient (DSC) and Hausdorff distance (HD95). Higher DSC and lower HD95 scores indicate higher levels of agreement between the two segmentations. 30 randomly selected MRI scans were segmented by a separate radiologist and compared to the corresponding reference segmentations to assess interobserver agreement. To determine intraobserver agreement, 20 randomly selected MRI scans were segmented a second time after a wash-out period of two weeks.

Radiomics and DL methods are discussed below. The data from each center was grouped into four trial sets for testing while data from the remaining centers is used for cross-validation (Table 7). In order to simulate a real scenario, the test set consists of total data from one or two centers in each trial, which results in a different amount of test set data in each trial. These sets are referred to as Trial 1 (T1), Trial 2 (T2), Trial 3 (T3), and Trial 4 (T4). The groupings of studies across Trials were chosen such that there is balanced representation of low-, and high-risk studies across the training and test sets. To ensure robust and comprehensive validation, models from each trial were evaluated using a different center’s data for testing.

### Radiomics-only analysis

We conducted a 2D and 3D radiomics analysis (Fig. 6-B). Images were resized to achieve an isotropic voxel size of 1 mm^3^ (3D analysis) or an isotropic pixel size of 1 mm^2^ (2D analysis), using linear interpolation. An N4 bias field correction was applied to reduce low-frequency variations in the acquired signals^43^. Intensity values were normalized using the min-max normalization technique^44^. Then, radiomic features were extracted from the preprocessed images using in-house software and the package *collageradiomics* developed with Python^45-48^. For the 3D analysis, 763 radiomic features were extracted within the entire volume of the cyst. For the 2D analysis, 447 features were extracted from axial plane slices. Features were extracted from six radiomic families. To capture spatial properties of pixel intensities were used the Raw (original intensity values) and Gray (image was fileted with median, mean, std and range filters) families. To capture edge-related features were used Gradient (image was filtered with Sobel and gradient-like kernel filters) and Law’s (images were filtered by local specific masks) families^49-51^. Additionally, Haralick and CoLlAGe feature families were used to characterize the co-occurrence matrices (GLCM) ^46,47^. The window sizes used to construct the GLCM matrices were w = 3×3, w = 5×5, and w = 7×7, and the number of gray levels was set to 4, 8, 16, 32, and 64 (See detailed description of radiomic families in Table S1 of supplementary material). The features were calculated inside the entire region of interest (the cyst) and then each feature was represented by four statistical measures: median, standard deviation, skewness, and kurtosis. Using the training set of each trial, a Spearman correlation threshold of 0.6 was applied to remove the most correlated features. Then, a 5-fold cross-validation scheme with 50 iterations was applied to select the best features applying the Maximum Relevance Minimum Redundancy (mRMR) algorithm and training a Random Forest (RF) model^52^. After cross-validation, features selected in at least 70% of the iterations were picked up. Finally, a RF was trained with the entire training set and tested with the hold-out set.

### Deep learning-only analysis

The DL experiment was done in two parts (Fig. 6-C). First, we applied 5-fold cross-validation on the entire dataset from all centers to select the best performing model. We assessed the performance of six advanced convolutional neural networks (CNNs): EfficientNet-B0^29^, MobileNet-V2^31^, ResNet-34^28^, ResNet-50^28^, ShuffleNet-V2^30^, and DenseNet-121^27^. ROIs were cropped based on whole pancreas segmentation published in Zhang et. al., 2025^25^. Images were shuffled and resized to 96×96×96 for training. Models were trained using stochastic gradient descent (SGD) with a momentum of 0.9 and a batch size of 2, for a total of 200 epochs. The initial learning rate was set at 0.001 and decreased by a factor of 10 every 30 epochs. A 5-fold cross-validation process was applied to enhance result robustness. In the second part, we split the dataset by center, based on the four trial sets as described in Table 7. Each set was then used to train the best performing DL model from part one using the same parameters.

### Radiomics-deep learning fusion algorithm

Our radiomics-DL fusion algorithm was developed by fusing decision probabilities of the radiomics Random Forest classifier with the best performing CNN in the DL-only analysis, DenseNet121 ^27^ (Fig. 6-C). Radiomics feature refinement was done by applying a 5-fold cross-validation and selecting radiomic features with a Spearman correlation coefficient below 0.6 to minimize redundancy. Both the radiomics-based Random Forest model and the deep learning model were retrained using the same training set, which was consistently split to ensure comparability. After training, the predicted probabilities from both models were fused, and the combined output was evaluated on the validation and test sets. For decision-level fusion, the probability outputs from both the DenseNet121 and Random Forest models were combined. Inspired by our earlier work (Yao et al 2023^23^), we applied an exact sample to the fusion method and found the best hyperparameter with grid search on fivefold cross validation^18^. Two hyperparameters were introduced in the fusion method: the threshold *t* and the weight *k.* If the radiomics prediction exceeds the threshold *t*, the final model output was solely based on the radiomics prediction, and the DL prediction was discarded. Otherwise, the fusion output was a weighted combination of the predictions from the radiomics-based model and the DL model, with the weight of the radiomics-based model set to *1-k* and weight of the DL model set to *k.* The fusion pipeline was conducted twice, using either 2D or 3D radiomic features. Weighted averages were calculated because of differences in the number of subjects represented in each center. A visualization of our fusion pipeline is provided in Fig. 6-C.

### Radiologist Visual Scoring

Images were visually scored by three independent, expert radiologists [GD, FB, YBT] using the imaging features of the Kyoto Criteria^2^. Cysts were given the label of no risk, low risk, or high risk according to radiological assessment. The radiologists were not told that the cysts were confirmed IPMN to emulate real-life, initial cystic lesion evaluation. Additionally, the radiologists were blinded to the subject’s clinical information, utilized only T2W and contrast-enhanced T1W sequences, and did not have access to previous imaging. 13 cases from the study cohort were excluded from this analysis because T1W images were not available (n = 347). A pairwise assessment of weighted kappa statistics was calculated to evaluate agreement between raters. Sensitivity and specificity were calculated to evaluate the accuracy that the radiologists identified a high-risk lesion correctly.

## Figures and Tables

**Table 1:** Comparison of rater performance in visual scoring of IPMN by the imaging features of the Kyoto Criteria^2^. Sensitivity and specificity of each rater (n = 347), and a pooled sensitivity and specificity for subjects that received the same score by a majority of the raters (n = 337).

	Sens (%)	Spec (%)	Acc (%)
**Rater 1**	68.9	64.5	66.7
**Rater 2**	42.2	32.6	37.4
**Rater 3**	72.3	41.8	57.1
**Majority**	70.6	63.2	66.9

**Table 2:** Inter-rater comparison of accuracy and agreement in visual scoring of IPMN by the imaging features of the Kyoto Criteria.

	Acc (95% CI)	Weighted Kappa (95% CI)
**Rater 1 vs 2**	48.1 (42.7, 53.5)	0.33 (0.27, 0.39)
**Rater 2 vs 3**	61.7 (56.3, 66.8)	0.67 (0.59, 0.74)
**Rater 1 vs 3**	80.1 (75.5, 84.1)	0.47 (0.39, 0.53)

**Table 3:** Radiomics-only results for 2D and 3D analysis for each trial set.

**Testing - Random Forest (%)**	**F1**	**52.2 ± 6.0**	**60.4 ± 5.5**	**72.4 ± 4.1**	**61.8 ± 3.9**	**61.7 ± 5.0**	**60.3 ± 3.4**	**57.4 ± 4.5**	**66.5 ± 4.5**	**60.2 ± 3.6**	**61.1 ± 4.0**
	**Spec**	**46.2 ± 13**	**62.8 ± 8.7**	**75.0 ± 4.1**	**67.8 ± 6.6**	**63.1 ± 8.8**	**58.2 ± 5.7**	**54.5 ± 13**	**72.9 ± 6.9**	**70.6 ± 7.2**	**64.1 ± 8.5**
	**Sens**	**62.9 ± 13**	**65.0 ± 8.7**	**78.2 ± 7.5**	**77.0 ± 9.3**	**71.0 ± 9.0**	**68.7 ± 5.3**	**64.7 ± 12**	**70.4 ± 9.1**	**71.1 ± 7.0**	**68.7 ± 8.8**
	**PPV**	**45.9 ± 6.9**	**57.0 ± 5.5**	**67.7 ± 3.2**	**52.1 ± 4.5**	**55.7 ± 5.2**	**53.9 ± 3.4**	**52.5 ± 6.1**	**63.8 ± 5.1**	**52.7 ± 5.1**	**55.7 ± 5.0**
	**Acc**	**53.1 ± 5.3**	**63.7 ± 4.5**	**76.3 ± 2.9**	**70.6 ± 3.4**	**65.9 ± 4.1**	**62.6 ± 3.6**	**58.9 ± 3.8**	**71.9 ± 3.6**	**70.8 ± 4.0**	**66.1 ± 3.8**
	**AUC**	**46.7 ± 5.4**	**61.9 ± 4.2**	**77.0 ± 3.5**	**79.6 ± 2.5**	**66.4 ± 4.0**	**59.2 ± 2.5**	**58.8 ± 2.9**	**73.5 ± 3.3**	**74.6 ± 2.3**	**66.5 ± 2.8**
**Cross-Validation - Random Forest (%)**	**F1**	62.9 ± 2.1	63.0 ± 1.9	62.2 ± 1.8	62.8 ± 2.0	**62.7 ± 1.9**	63.9 ± 1.7	62.9 ± 1.9	59.8 ± 2.2	62.9 ± 2.3	**62.4 ± 2.0**
	**Spec**	68.9 ± 4.7	69.7 ± 4.5	68.8 ± 4.9	68.1 ± 4.8	**68.9 ± 4.7**	69.8 ± 4.3	68.3 ± 4.6	67.0 ± 4.5	68.2 ± 4.2	**68.3 ± 4.4**
	**Sens**	67.7 ± 4.2	67.5 ± 4.4	66.6 ± 4.5	66.1 ± 4.7	**67.0 ± 4.5**	68.7 ± 4.2	68.3 ± 4.1	64.2 ± 4.6	66.4 ± 5.2	**66.9 ± 4.5**
	**PPV**	58.9 ± 2.9	59.3 ± 2.6	58.6 ± 2.8	60.0 ± 2.7	**59.2 ± 2.7**	59.9 ± 2.6	58.5 ± 3.0	56.2 ± 2.7	60.1 ± 2.1	**58.7 ± 2.6**
	**Acc**	68.4 ± 2.1	68.9 ± 1.8	67.9 ± 1.9	67.3 ± 1.8	**68.1 ± 1.9**	69.3 ± 1.7	68.3 ± 2.0	65.9 ± 2.0	67.4 ± 1.5	**67.7 ± 1.8**
	**AUC**	72.6 ± 1.5	72.7 ± 1.2	71.8 ± 1.6	71.2 ± 1.5	**72.1 ± 1.5**	73.5 ± 1.3	72.2 ± 1.7	69.0 ± 1.8	71.5 ± 1.5	**71.6 ± 1.6**
	**Features**	28	30	20	30	**Average**	28	30	30	30	**Average**
	**Trial**	T1	T2	T3	T4		T1	T2	T3	T4	
		**2D Radiomics**					**3D Radiomics**				

**Table 4:** Deep Learning results and standard deviation of IPMN cyst malignancy risk stratification in 5 folds cross-validation^27-31^.

	AUC (%)	Acc (%)	Sens (%)	Spec (%)
**DenseNet121**	73.3 ± 7.9	68.0 ± 7.7	46.5 ± 23	82.7 ± 6.5
**Mobilenetv2**	73.0 ± 2.9	66.6 ± 2.3	N/A	N/A
**ResNet34**	73.1 ± 4.7	68.5 ± 4.4	N/A	N/A
**ResNet50**	71.8 ± 6.3	66.0 ± 6.1	N/A	N/A
**ShuffleNet-V2**	66.6 ± 5.7	61.3 ± 1.7	N/A	N/A
**EfficientNet-B0**	68.1 ± 1.1	65.6 ± 6.0	N/A	N/A

**Table 5:** Radiomics-deep learning fusion algorithm results. 2D and 3D radiomic features were fed into DenseNet121 in 5 cross-validation on 4 different trials^27^.

Cross Validation (%)	Spec	60.9 ± 9.6	62.9 ± 14.3	77.6 ± 17.3	72.2 ± 11.6	68.4 ± 7.8	100.0 ± 0.0	98.4 ± 2.0	98.3 ± 2.3	96.3 ± 4.6	98.3 ± 1.5
	**Sens**	81.2 ± 10.2	78.5 ± 12.3	63.9 ± 13.1	77.6 ± 17.4	**75.3 ± 7.8**	98.5 ± 1.9	100.0 ± 0.0	98.5 ± 3.0	95.1 ± 3.6	**98.0 ± 2.1**
	**Acc**	68.7 ± 2.6	68.7 ± 5.9	71.5 ± 4.9	74.9 ± 3.2	**71.0 ± 3.0**	99.4 ± 0.8	99.1 ± 1.2	98.5 ± 1.4	96.5 ± 1.1	**98.4 ± 1.3**
	**AUC**	73.3 ± 2.9	74.6 ± 4.7	73.9 ± 2.6	75.6 ± 4.2	**74.3 ± 3.6**	77.1 ± 3.2	74.0 ± 6.1	70.0 ± 4.8	72.5 ± 2.2	**73.4 ± 4.1**
**Testing (%)**	**Spec**	66.7 ± 12.9	66.7 ± 24.2	54.3 ± 24.6	75.5 ± 16.9	**67.8 ± 7.9**	63.3 ± 4.1	62.7 ± 13.1	35.7 ± 10.1	77.3 ± 5.0	**63.3 ± 15.4**
	**Sens**	35.3 ± 9.8	46.0 ± 28.4	77.1 ± 10.2	63.7 ± 13.9	**57.9 ± 14.4**	56.5 ± 6.0	57.0 ± 14.0	81.0 ± 9.0	60.4 ± 7.6	**63.2 ± 9.1**
	**Acc**	48.3 ± 2.2	54.9 ± 6.9	68.0 ± 7.1	67.3 ± 4.9	**61.6 ± 7.9**	59.3 ± 3.4	59.4 ± 3.3	62.9 ± 7.3	65.6 ± 3.8	**62.7 ± 2.8**
	**AUC**	47.1 ± 1.7	61.7 ± 3.8	77.5 ± 5.5	79.0 ± 1.6	**69.2 ± 2.9**	59.3 ± 3.6	63.3 ± 2.3	67.6 ± 4.7	74.7 ± 2.2	**68.3 ± 2.9**
	**Trial**	T1	T2	T3	T4	**Weighted Average**	T1	T2	T3	T4	**Weighted Average**
		**2D Radiomic Features**					**3D Radiomic Features**				

**Table 6:** Breakdown of IPMN subtype in the Low and High-Risk groups.

	BD-IPMN (%)	Mixed & MD-IPMN (%)
**Low Risk**	155 (78.2)	62 (38.5)
**High Risk**	43 (27.7)	99 (61.5)
**Total**	198	161

**Table 7:** Centers used for testing and cross validation of the four trial sets used in the radiomics, DL, and radiomics-DL fusion analyses.

Trial	Cross Validation (N)	Testing (N)
**T1**	EMC, IU, MCF, NMH, & NYU (330)	AHN & MCA (29)
**T2**	AHN, IU, MCA, MCF, NMH, & NYU (323)	EMC (36)
**T3**	AHN, EMC, MCA, MCF, NMH, & NYU (324)	IU (35)
**T4**	AHN, EMC, IU, MCA, MCF, & NMH (288)	NYU (71)

## Data Availability

Our MRI and corresponding excel file for risk status of the patients are available at OSF server (NIH supported data sharing platform) at https://osf.io/74vfs/.

## Code Availability Statement:

The underlying code for this study is available in GitHub and can be accessed via this link: https://github.com/Zilian4/IPMN-Radiomics-Plus-Deeplearning.
